# Neural signal data collection and analysis of Percept^™^ PC BrainSense recordings for thalamic stimulation in epilepsy

**DOI:** 10.1088/1741-2552/ad1dc3

**Published:** 2024-02-06

**Authors:** Zachary T Sanger, Thomas R Henry, Michael C Park, David Darrow, Robert A McGovern, Theoden I Netoff

**Affiliations:** 1 Department of Biomedical Engineering, University of Minnesota, Minneapolis, United States of America; 2 Department of Neurosurgery, University of Minnesota, Minneapolis, United States of America; 3 Department of Neurology, University of Minnesota, Minneapolis, United States of America

**Keywords:** deep brain stimulation, anterior nucleus of thalamus, DBS, Percept, SenSight, epilepsy, closed-loop neuromodulation

## Abstract

Deep brain stimulation (DBS) using Medtronic’s Percept^™^ PC implantable pulse generator is FDA-approved for treating Parkinson’s disease (PD), essential tremor, dystonia, obsessive compulsive disorder, and epilepsy. Percept^™^ PC enables simultaneous recording of neural signals from the same lead used for stimulation. Many Percept^™^ PC sensing features were built with PD patients in mind, but these features are potentially useful to refine therapies for many different disease processes. When starting our ongoing epilepsy research study, we found it difficult to find detailed descriptions about these features and have compiled information from multiple sources to understand it as a tool, particularly for use in patients other than those with PD. Here we provide a tutorial for scientists and physicians interested in using Percept^™^ PC’s features and provide examples of how neural time series data is often represented and saved. We address characteristics of the recorded signals and discuss Percept^™^ PC hardware and software capabilities in data pre-processing, signal filtering, and DBS lead performance. We explain the power spectrum of the data and how it is shaped by the filter response of Percept^™^ PC as well as the aliasing of the stimulation due to digitally sampling the data. We present Percept^™^ PC’s ability to extract biomarkers that may be used to optimize stimulation therapy. We show how differences in lead type affects noise characteristics of the implanted leads from seven epilepsy patients enrolled in our clinical trial. Percept^™^ PC has sufficient signal-to-noise ratio, sampling capabilities, and stimulus artifact rejection for neural activity recording. Limitations in sampling rate, potential artifacts during stimulation, and shortening of battery life when monitoring neural activity at home were observed. Despite these limitations, Percept^™^ PC demonstrates potential as a useful tool for recording neural activity in order to optimize stimulation therapies to personalize treatment.

## 1. Introduction

Deep brain stimulation (DBS) is United States Food and Drug Administration (FDA) approved for the treatment of essential tremor [1, 2], Parkinson’s disease (PD) [3-6] and epilepsy [7-10]. Traditional DBS is open-loop where stimulation parameters are decided during a clinical visit and remain constant between visits regardless of ongoing physiological changes within the brain. Medtronic’s Percept^™^ PC is an implantable pulse generator (IPG) able to record neural signals from the same lead, through contacts adjacent to the stimulation contacts. This enables the recording of neural signals originating from the same cerebral area as that being stimulated. As a result, closed-loop stimulation paradigms are possible with this device. Creative applications of Percept^™^ PC’s closed-loop capabilities are already emerging in the field of neuromodulation. Yohann Thenaisie’s group in 2021 used Percept^™^ PC to access and capture gait and medication-induced beta power local field potential (LFP) changes in PD and dystonia patients [5]. Jimenez–Shahad in 2021 reviewed Percept^™^ PC as a closed-loop neuromodulation tool for movement disorders, noting the unique functionality over other currently available systems [4]. Here we showcase example data from Percept^™^ PC used in a refractory epilepsy patient population and further discuss the features and engineering concepts embedded within device functionality of Percept^™^ PC. While further development of DBS technology is necessary, fully harnessing current device capabilities could bring improvements in therapy outcomes.

Percept^™^ PC has sufficient stimulus artifact rejection shown below which allows for capture of neural recordings even during stimulation. It can be used to stream neural signals in the clinic at a high sampling rate and at home in 10 min averages of power in a user-determined power band for up to 2 months between data downloads. This technology provides many opportunities for optimizing open-loop therapies based on neural feedback and fully closed-loop therapies. We have been using Percept^™^ PC in a clinical study to optimize stimulation settings for patients with epilepsy based on their neural activity at different stimulation settings. As engineers and neurophysiologists who have used Percept^™^ PC, our intent here is to share our observations and further explain the capabilities of the device to help answer potential questions that may arise during its use as a clinical research tool.

The target audiences of this paper are neurologists, neurosurgeons, device representatives, technicians, and scientists that are using Percept^™^ PC clinically or as a research tool. We will explain the features of Percept^™^ PC, the RAW data processing and filtering methods, and some of the filtering characteristics while highlighting some of the improvements of Percept^™^ PC over older versions. The focus will not be on the neural signals but instead on the characteristics of the recorded signals imparted by Percept^™^ PC. The data shown throughout this paper are examples collected from seven patients in our ongoing epilepsy clinical trial #NCT05493722 [11] which is investigating the use of Percept^™^ PC to optimize stimulation settings in patients with drug refractory epilepsy. More information on the patient population can be found in the appendix.

### 1.1. Percept^™^ PC hardware

Typically, DBS patients are implanted with subcortical leads connected to the Percept^™^ PC IPG through an extension. Unlike most IPGs, Percept^™^ PC can record neural signals while applying a stimulus. Sensing hardware used within Percept^™^ PC IPG is based upon hardware used in the Medtronic Summit research IPG. The Summit’s sensing integrated circuit (IC) is reported to have a 100 nV rt^−1^ Hz minimum signal resolution [12] with an average noise floor over the frequency range from 0.05 to 100 Hz of 0.98 uVrms [12-14]. However, Jimenez—Shahed reported the Percept^™^ PC sensing noise floor to be <300 uV rt^−1^ Hz and the input sensing range to be 0.55–400 uVrms [4] while Goyal *et al* reported the Percept^™^ PC sensing noise floor of 150 nV rt^−1^ Hz [3]. Percept^™^ PC’s Analog to Digital Converter (ADC) can convert an analog neural signal digitally with 16 least significant bits (LSB) with a 250 gain amplified voltage to a range of ±1.2 volts [15]. The resulting ADC analog input range is then ±4.8 mV. The minimum ADC resolution is then 146 nV, calculated using ADC Input Range = 2^LSB^ * ideal input resolution [16].

**Figure 1.**
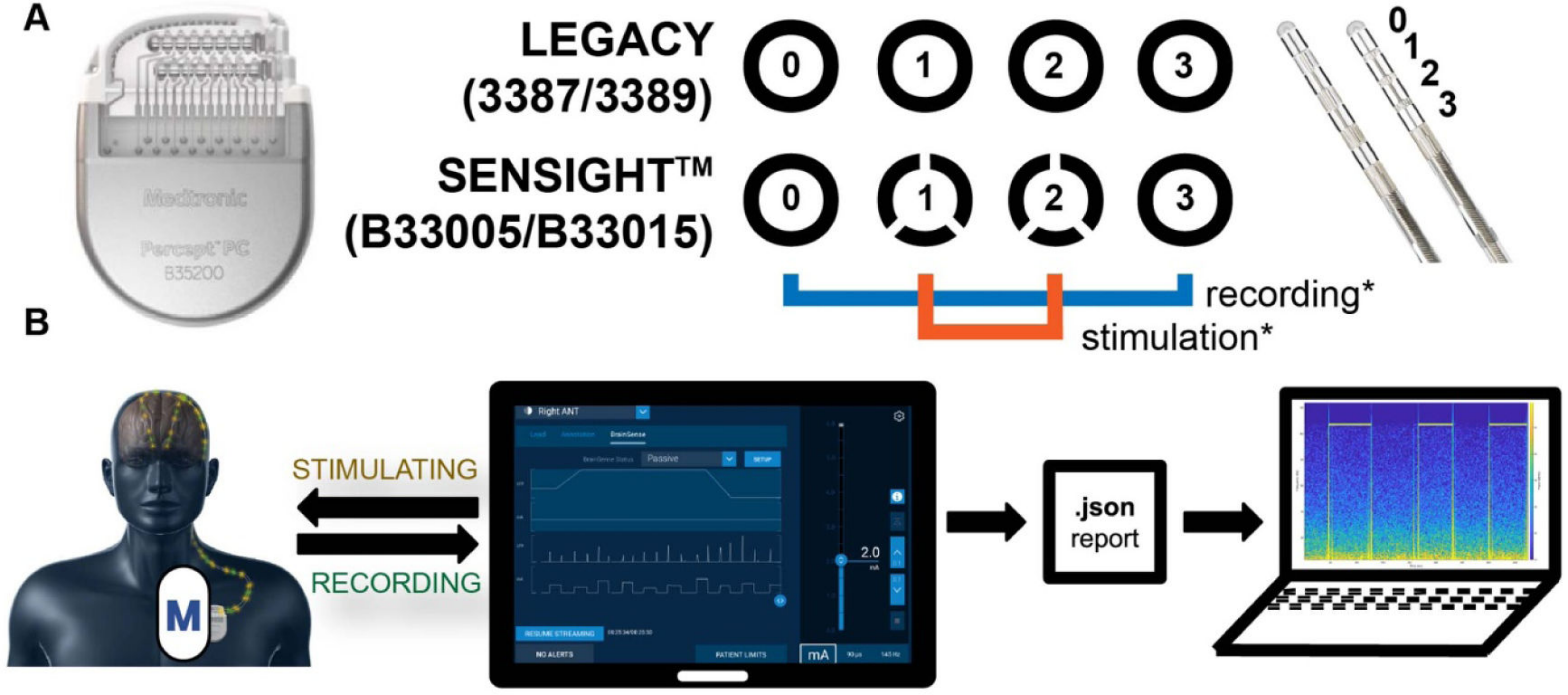
Medtronic’s Percept^™^ PC neurostimulation and recording system. (A) IPG and leads. Within our trial, the stimulation lead configuration is shown in orange, and the recording configuration is shown in blue. SenSight^™^ electrodes allow for additional directional stimulation configurations through the middle rings, which are divided into three contacts. (B) The clinical physician tablet connects to the patient device via the clinical telemetry module (device marked M) and is used to program and capture data from the IPG. A data report containing the LFP, long-term recordings, and other metadata can be exported as a ‘.json’ report for further analysis. Device, lead, and sensing images provided by Medtronic, Inc. ^©^2023 Medtronic. All rights reserved. Used with the permission of Medtronic.

Both legacy (3387/3389) and SenSight^™^ (B33005/B33015) leads are compatible with the Percept^™^ PC IPG and their electrode layouts are shown in figure 1. SenSight^™^ leads have been reported to show a 57% improvement in tip stability over Legacy leads [17]. Shown in figure 1(A), SenSight^™^ leads differ from Legacy leads in that the two middle ring contacts (1 and 2) are each divided into three contacts, which allows for current steering and other stimulation customization. This feature was not utilized within this study. Patients in our study were programmed with a cathode stimulation configuration at electrode sites 1 and 2 while using the IPG case as the anode. Bipolar recordings were measured between surrounding electrode sites 0 and 3. The ‘0’ electrode is the deepest or most distal contact on the lead.

In the clinic, physicians interact with the Percept^™^ PC device via a Medtronic tablet. This tablet allows for the visualization and recording of a multitude of measurements, including impedance, patient-reported events, device-reported events, power spectra filter adjustments, and stimulation settings and parameters. Data can be exported for analysis in a JavaScript Object Notation ‘.json’ report (see figure 1(B)).

### 1.2. Percept^™^ PC BrainSense setup in clinic

During a patient visit in the clinic, a clinical telemetry module is placed over the IPG to establish a wireless connection from the IPG to the tablet. Upon connection to the patient IPG, an initial check is run to ensure that the impedance of the patient’s electrodes is within safe ranges. This measure ensures proper stimulation delivery and verifies that the lead connection is functioning normally. The IPG is equipped to ramp stimulation on and off for patient comfort. This ramping generates electrical noise, as will be shown in the results, and to minimize artifacts and improve the speed of changing settings, we often disable the soft start feature when testing settings and enable it during therapy. Therapeutic stimulation is delivered intermittently (‘cycling’ is often 1 min on and 5 min off) for epilepsy and continuously for Parkinson’s disease. For in-clinic recordings of the neural response to stimulation, we set the device to continuous stimulation. The treating physician determines which electrodes are used as anodes and cathodes. The amplitude of the current can be independently programmed for each electrode contact due to Percept^™^ PC’s 16 independent sources and sinks [18].

The Percept^™^ PC BrainSense Streaming [19] feature allows for continuous recording from the electrodes surrounding the stimulation electrodes. The recording electrodes span the stimulus electrodes to sample at opposite sides of the dipole for maximum stimulus artifact rejection prior to amplification and digitization of the signal. In the clinic, Percept^™^ PC can record at 250 Hz. At home, Percept^™^ PC can record 10 min averages of the power measured in a 5 Hz frequency band of interest which can be selected by the physician. BrainSense can be activated following impedance verification and a short recording to measure the power spectrum of the recorded neural data to facilitate selection of the center frequency for at-home recordings.

The stimulation amplitude, frequency, and pulse width are limited to safety bounds and settings that minimize stimulation artifacts and harmonics that interfere with data acquisition. The device is designed to prevent potential tissue damaging stimulus waveforms and is limited to amplitude, pulse width, and frequency rates with a charge density less than 30 uC cm^−2^/phase on an electrode surface area of 0.06 cm^2^ before prompting the user through the device programmer that they are exceeding safety limits [20]. Additionally, stimulation parameter ranges can be limited by the device for better BrainSense recordings. We observed on the physician programming tablet that frequency ranges within BrainSense while stimulating were limited to frequencies of 55, 85, 110, 125, 145, 165, and 180 Hz with respective pulse width and amplitude limits following the safety limits above. The maximum current amplitude has been reported as 25.5 mA for Summit [12] and 25.5 mA at 500 Ω for Activa PC [20]. When the minimum frequency and pulse width of 55 Hz and 20 *μ*s were selected using the physician programming tablet BrainSense streaming demo mode, the maximum current amplitude allowed was 12.9 mA regardless of safety warnings.

### 1.3. Percept^™^ PC data formatting

The Percept^™^ PC system can stream data to the tablet, which can be exported with optional deidentification as a file through the clinical programmer app for further analysis. A 30 min recording exported as a ‘.json’ report is typically less than 20 Mb in size. To load a ‘.json’ data report into MATLAB as a data structure, MATLAB’s ‘jsondecode’ [21] function will return a data structure given the report’s file path. Within this structure, the in-clinic recordings are stored in the ‘BrainSenseTimeDomain’ structure. Within BrainSenseTimeDomain, raw LFP data are stored within ‘TimeDomainData’ and each entry corresponds to a recording separated by a user-initiated pause in data recording or a change in programmed stimulation frequency while recording. Additional metadata about the raw data are shown in other entries within this data structure. At-home recording data can be accessed under the ‘LFPTrendLogs’ data structure within the ‘DiagnosticData’ data structure. At-home 10 min average power recordings are stored as ‘LFP’ in the structure, and each entry has a ‘DateTime’ stamp. Patient-reported at-home events can be synced with these timestamps for at-home timeline-plotted LFP data and noted event activity. We have cited multiple tools which can be accessed to process Percept^™^ PC exported data [5, 22-24].

### 1.4. Percept^™^ PC filtering characteristics

The Percept^™^ PC IPG filter design is based on the Summit^3^ IPG. The recordings from Percept^™^ PC are in the time domain, but it is often useful to understand the neural signals and filtering characteristics of the device by studying them in the frequency domain. Time series data can be transformed to the frequency domain with the Fourier transform. The Fourier transform compares the recorded signal to pairs of sine and cosine waves at different frequencies. The Fourier-transformed signal at a particular frequency is determined by the sum of the correlation of that signal with the sine and cosine functions squared. The power of the Fourier-transformed signal is represented as the power spectral density. The effects of filters on an input to produce a revised output at different frequencies is often illustrated in the frequency domain through the power spectrum.

One property of the Fourier transform is that power is preserved at all frequencies. When analyzing digitized data sampled discreetly in time, the highest frequency that can be visualized by the discrete Fourier transform is half the sampling rate, called the Nyquist frequency. Any power above Nyquist is folded down into the lower frequencies and added, thereby corrupting the lower-frequency power estimates. As a result, if stimulating at any frequency above the Nyquist frequency, (125 Hz for a 250 Hz sampling rate), the signal reflected at Nyquist is aliased into the lower frequencies (e.g. 145 Hz is 20 Hz greater than 125 Hz and therefore aliases at 125–20 Hz =105 Hz). Therefore, it is important to remove signals above Nyquist before digitizing the signal.

Filters in Percept^™^ PC reduce system and environmental noise to better isolate the neural activity of interest. The device contains multiple analog filters that are implemented in the hardware that smooth the data (low pass) or remove low-frequency drift or artifacts (high pass) prior to digitization of the signal. Analog filters can be repeated in series to create a sharper transition between the frequencies that are passed and those that are removed; each repeat of the filter increases the ‘order’ to the filter. Digital filters are applied to the signal after it has been digitized and have the advantage of being easily programmable for adjustment by the user to further clean the data prior to display or storage.

**Figure 2.**
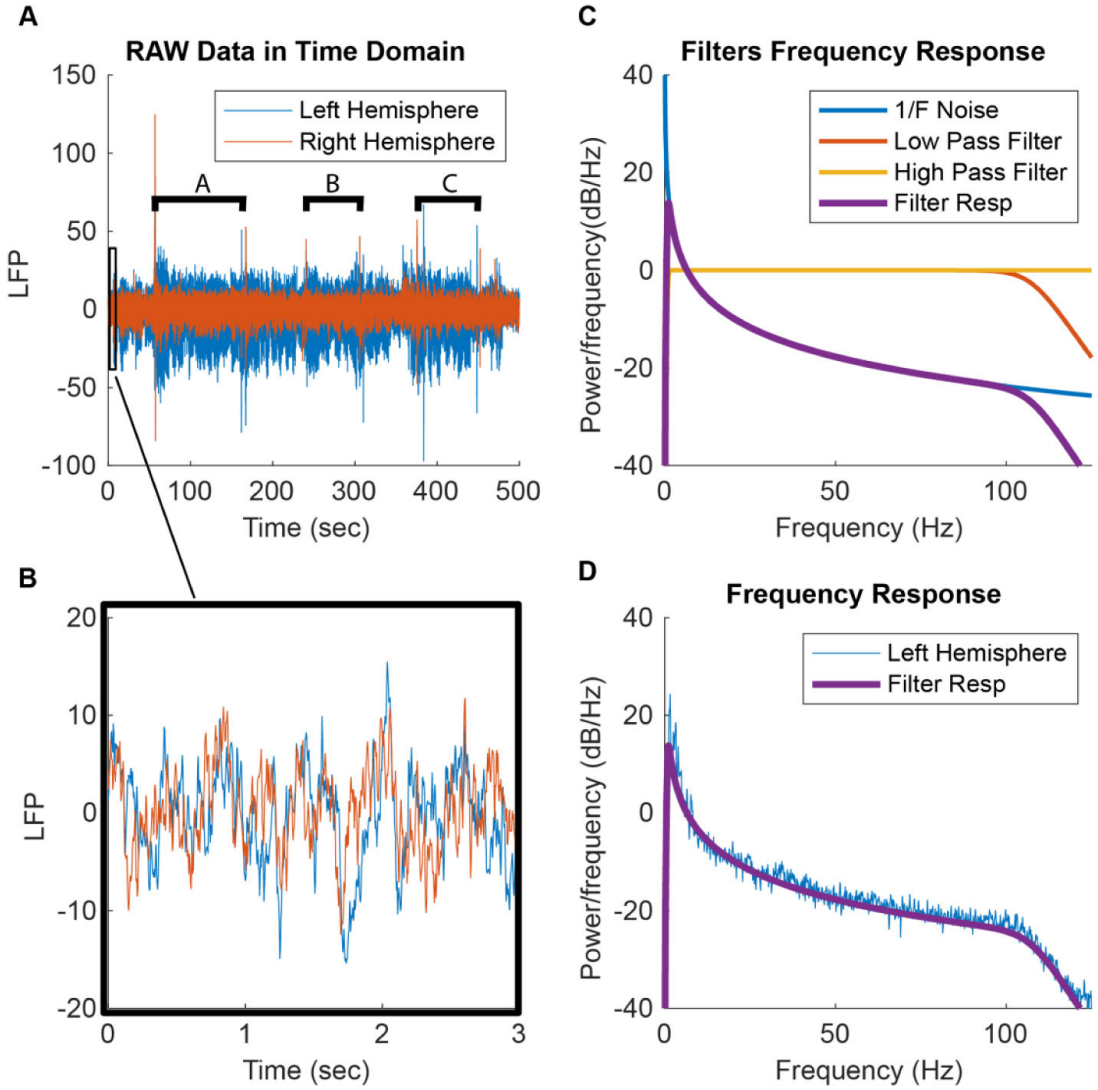
Percept^™^ PC temporal and spectral features. (A) Raw left and right hemisphere LFP data from the anterior nucleus of the thalamus (ANT) from Patient 005 (info in Appendix) with and without stimulation at 3 different settings (A-90 *μ*s, B-120 *μ*s, and C-60 *μ*s, all at 145 Hz). (B) Three seconds of ‘stimulation off’ raw LFP data. (C) Idealized neural activity represented by 1 frequency^−1^ noise, the idealized high and low pass filters in Percept^™^ PC, and the combination of all three filters to show the device’s estimated ideal total filter response. (D) Estimated idealized total filter response shown in 2 C compared to the power spectrum of ‘stimulation off’ data before stimulation shown in the neural recording of the left hemisphere shown in 2 A.

The hardware of the Percept^™^ PC IPG is similar to that of the Summit IPG [12, 25] and includes a 1st-order analog high pass filter at 0.5 Hz to remove low-frequency noise, a 1st- and 3rd-order analog low pass filter at 100 Hz to reduce aliasing and stimulation artifacts, which will be described later, and a 4th-order digital elliptic filter to further reduce digitization noise and aliasing and stimulation artifacts [12]. Figure 2 shows raw data recorded while the patient was at rest and the theoretical spectrum calculated using an estimate of the electronic noise and the filtering characteristics. It can be seen that much of the power spectrum shape is explained by a pink noise model (power(frequency) = 1 f^−1^) and the high and low pass filters. Activity deviating from this shape can be generated by movement artifacts, system noise, environmental noise, or neural activity. Methods have been developed to remove this underlying filter response and 1 f^−1^ noise to focus on the contributions of the neural signal [10].

### 1.5. Time-frequency analysis of Percept^™^ PC BrainSense data

**Figure 3.**
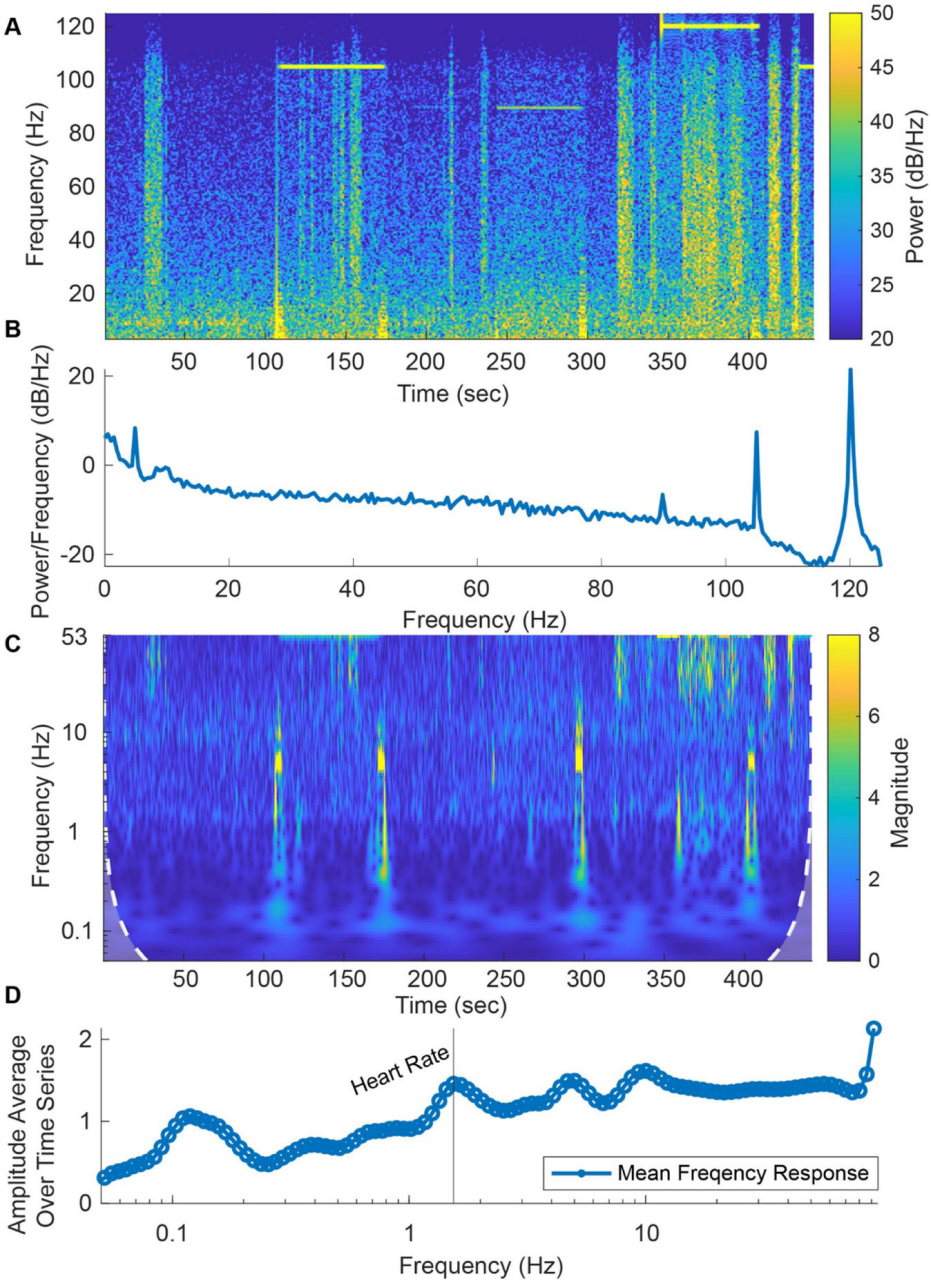
Spectrogram vs. Wavelet Time-Frequency Analysis of BrainSense Data. (A) Spectrogram of Patient 001 in-clinic raw LFP recordings containing one-minute stimulation and one-minute baseline recording intervals for the stimulation settings 120 Hz, 145 Hz, and 160 Hz with a 90 *μ*s pulse width. (B) Power spectrum showing the 120 Hz stimulation artifact and aliased artifacts at 105 Hz (from 145 Hz) and 90 Hz (from 160 Hz). Fourier analysis provides greater high-frequency resolution (10–125 Hz) across time. (C) Morlet wavelet analysis scalogram of the raw dataset used in 3 A. (D) Average frequency response across time. Wavelet analysis windowing allows for great low frequency resolution (1–10 Hz) across time. Heart rate (HR) is marked assuming an average rate of 60–100 beats per minute.

The Fourier transform treats the data as time invariant, meaning that the neural activity at the beginning of the recording is no different than that at the end of the recording. However, neural activity is constantly changing, and therefore, it is often better to represent the data in a time-frequency analysis. Time-frequency analysis can be calculated using a short-time Fourier transform or spectrogram in which the power spectrum is shown in short windows over time. Examples of spectrograms are shown in figures 3(C) and 5(B). The drawback of this method is that the resolution between 1 and 1.5 Hz is the same as that for 100–100.5 Hz.

An alternative to the Fourier transform is a wavelet transform, which uses a ‘wavelet’ instead of sines and cosines. An example of a wavelet is the Morlet wavelet, which uses a sine wave tapered by a gaussian window. Unlike the Fourier transform, the wavelet transform scales the wavelet to varying lengths, resulting in a time-frequency analysis where the temporal resolution and frequency resolution change as a function of the scaling. The wavelet transform around low frequencies has high frequency resolution but low temporal resolution, and at high frequencies, the temporal resolution is high while the frequency resolution is low. This provides a helpful balance between time and frequency resolutions for time varying neural signals. The magnitude scalogram in figure 3 displays the lower temporal resolution at low frequencies and higher temporal resolution at higher frequencies.

Comparison of the spectrogram and magnitude scalogram for the same data obtained from Patient 001 while applying three different stimulation settings shows that each frequency analysis method has its advantages. The spectrogram (here calculated using MATLAB’s spectrogram function) is better for analyzing high frequency and narrow band activity, such as the stimulation artifacts (figure 3(A)). In the spectrogram, the stimulation artifact at 120 Hz and the higher frequency stimulation artifacts aliased to lower frequencies (145 Hz aliased to 105 Hz and 160 Hz aliased to 90 Hz) can be seen (figures 3(A) and (B)). However, the magnitude scalogram calculated using the Morlet wavelet transform clearly demonstrates better low frequency activity resolution, such as theta and alpha oscillations, as shown in figure 3(C). Peaks in the average magnitude scalogram over time can be seen in the 1–10 Hz range of figure 3(D), where the heart rate peak can be detected.

### 1.6. Medtronic legacy vs. SenSight^™^ electrodes

**Figure 4.**
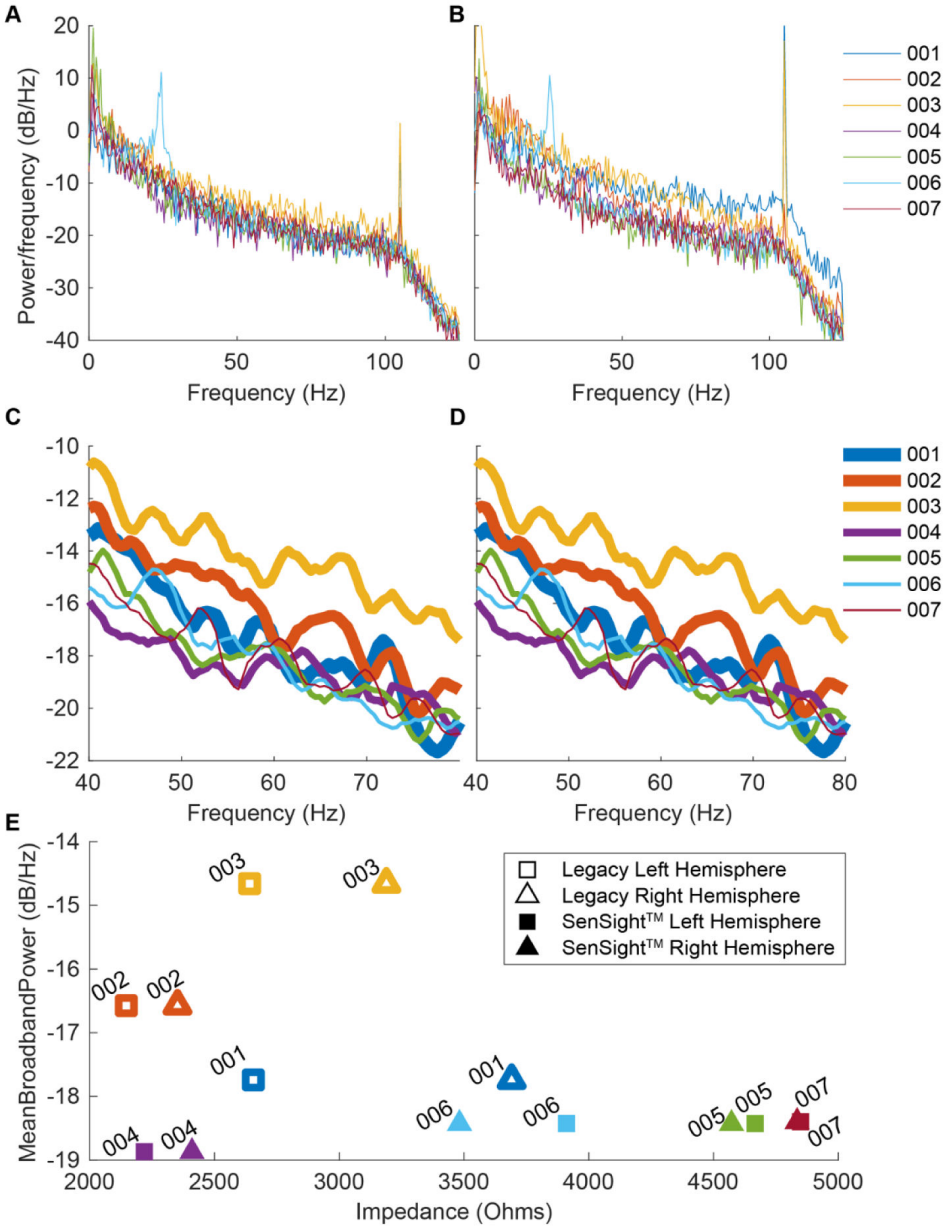
Medtronic Deep Brain stimulation electrode impedance characteristics. Power spectral density from baseline LFP data calculated from the left hemisphere (A) and right hemisphere (B) of each patient. Expanded baseline of the 40–80 Hz band to show predominantly 1 F^−1^ noise activity in the recording for the left (C) and right (D) hemispheres. (E) The mean frequency response from 40–80 Hz (shown in C and D) was plotted against the left and right 0–3 bipolar lead impedance for the Legacy and SenSight^™^ leads, where left hemisphere leads are shown with squares and right hemisphere leads with triangles, and hollow symbols represent Legacy leads and filled symbols represent SenSight^™^ leads. The 40–80 Hz mean frequency response and impedance were significantly correlated in the SenSight^™^ leads (*R* = 0.91, *p* = 0.0017) but not the Legacy leads (*R* = −0.1, *p* = 0.85).

In 2022, standard clinical practice within DBS for epilepsy switched from using Medtronic Legacy leads (3387/3389) to Medtronic SenSight^™^ leads (B33005/B33015). This switch brought about additional features and improvements in performance. Some patients within our study have received the Legacy leads while others have received the new SenSight^™^ leads, allowing us to compare the noise sensitivity of each lead to differences in impedance. Figure 4 shows the power spectrum and impedance of each electrode during baseline recordings. A large peak around ~22 Hz in the beta band can be seen in patient 006s baseline recording. Beta power in an ANT DBS implanted patient has been reported in a case study poster before [19, 26] motivating future biomarker discovery using Percept^™^ PC BrainSense in patients with epilepsy. In all the patient recordings, a 105 Hz peak, which is the aliased stimulation artifact of 145 Hz, even with a 0 mA stimulation amplitude, is observed. This peak is caused by the hardware switching of the stimulation, even when the stimulation amplitude is off. In order to avoid alpha and beta neural activity as well as higher frequency stimulation and stimulation frequency hardware switching artifacts, the power in the 40–80 Hz band was compared to the impedance of the electrodes to determine how the pink noise characteristics of the recordings depend on electrode impedance. In general, the Legacy electrodes had much lower impedances than the SenSight^™^ electrodes. The average power recorded with the Legacy electrodes was generally higher than that recorded with SenSight^™^ leads. We calculated the correlation between the impedance and the power and found a statistically significant correlation in the SenSight^™^ leads (*N* = 4, *R* = 0.91, *p* = 0.0017) but not in the Legacy leads (*N* = 3, *R* = −0.1, *p* = 0.85). The lower mean power recordings and higher impedances of the SenSight^™^ leads are likely due to the 57% stability improvements of this new design [17].

### 1.7. Percept^™^ PC artifact reduction and aliasing

A perennial problem of all electrophysiology recordings is the ubiquitous 60 Hz (for US and 50 Hz for Europe) electrical hum that pervades the recordings. This noise is almost absent in Percept^™^ PC BrainSense signals, because the recording system is completely contained in the body, which functions effectively as a Faraday cage to prevent these signals from reaching the recording electrodes or any other component in the recording system.

**Figure 5.**
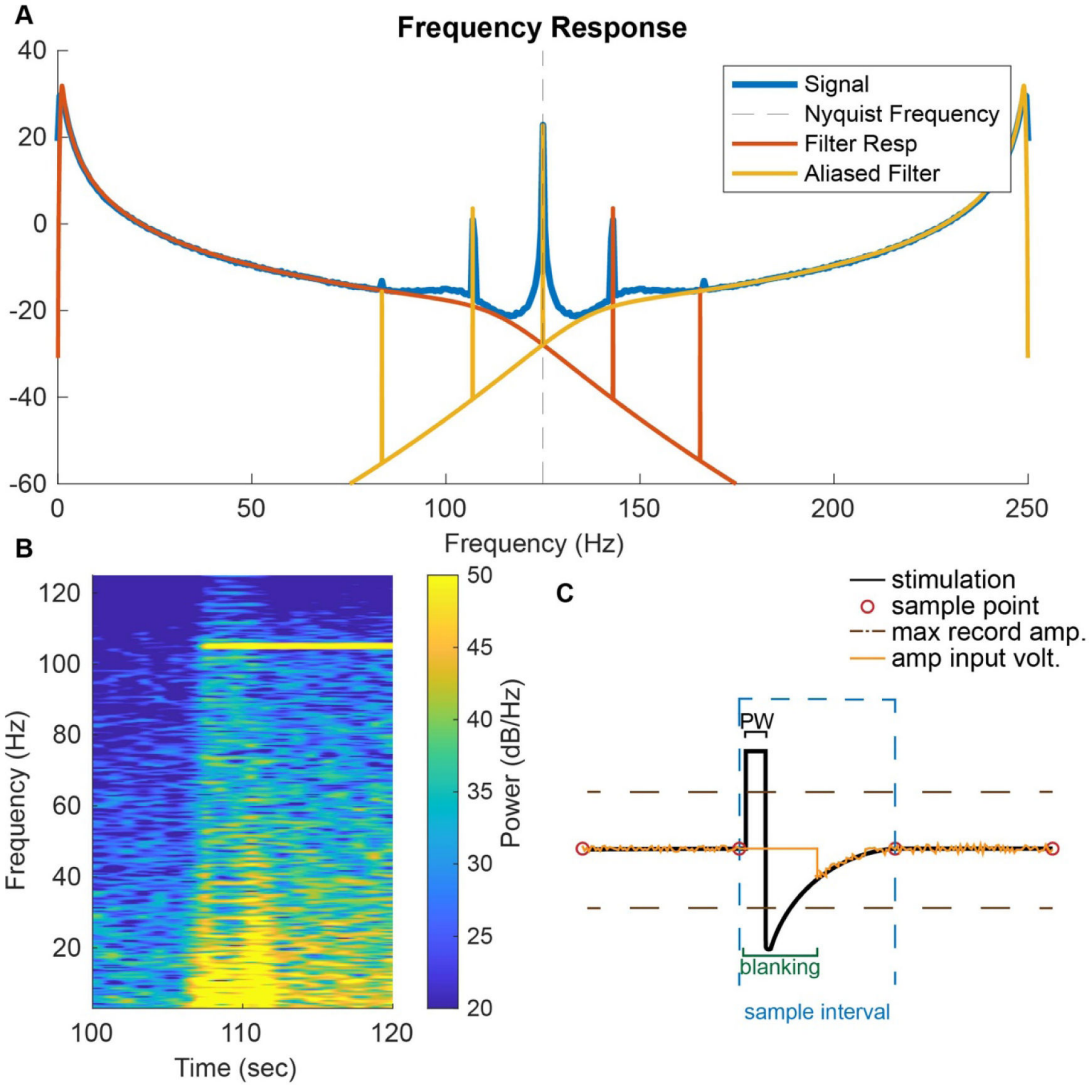
BrainSense data frequency activity and observed artifacts. (A) Frequency spectrum of data recorded from Patient 007, including 3 min of baseline and three one-minute segments with stimulation frequencies at 125 Hz, 145 Hz, and 165 Hz with 90 *μ*s pulse widths. The filter response and stimulation artifacts are shown in orange, but digitized signals at 125 Hz and greater in the power spectrum above the Nyquist frequency reflect at Nyquist as shown as the power spectrum is vertically flipped on the dashed line. The total response is a sum of the spectrum and reflected spectrum. (B) While ramping the stimulation amplitude by 0.1 mA steps using the ‘soft start’ feature, the amplifier creates a ‘banding’ artifact that is not neural. (C) Stimulation artifact removal through amplifier blanking. During stimulation, the amplifier input is shorted to prevent saturation and long recovery times of the amplifier. The stimulation in Percept^™^ PC is timed to arrive shortly after sampling to maximize the recovery time and minimize interference with the neural signal. The yellow trace shown is a simulated analog signal from an implanted lead prior to any digitization, amplification, or filtering. The black line represents the stimulation waveform while the red circles indicate the moments when the digitizer samples the data.

Stimulus artifacts do not necessarily show up in the recordings at the frequency of the stimulation but can be ‘folded’ into the sampling range through aliasing. Aliasing occurs in digitally sampled data where the highest frequency that can be recorded is half the sampling rate, called the Nyquist frequency. It is generally best practice to apply a low pass filter below the Nyquist frequency prior to digitizing the signal to prevent this. However, if a signal above Nyquist remains, the signal is folded down into lower frequencies. To explain this, figure 5(A) shows the ideal filter response with 1 F^−1^ pink noise generated in 2 A and the resulting idealized aliased response. Ideal, simulated stimulation artifacts were then added to the original which were subsequently aliased to overlay on a Percept^™^ PC recorded power spectral signal recording of baseline and stimulation delivered at 125 Hz, 145 Hz, and 165 Hz. Percept^™^ PC samples data at 250 samples a second, so the Nyquist frequency is 125 Hz. Therefore, the 165 Hz stimulation is aliased to be seen at 85 Hz. The low pass antialiasing filters built in Percept^™^ PC generally prevent any stimulation artifact above 170 Hz from confounding signals below 80 Hz.

The soft start feature in Percept^™^ PC can be customized to ramp stimulation by 0.1 mA/1, 2, 4, or 8 s, for the comfort of the patient. While testing settings, this feature adds time to the experiment as the amplitude slowly reaches the maximum. Given a 2 mA stimulation amplitude, this would add 20–160 s of additional recording time. The hardware switching of the amplitude also adds noise to the signal, causing a ‘banding’ effect shown between ~105–113 s, which can be seen in the power spectrum shown in figure 5(B). Therefore, during in clinic visits with BrainSense recordings, we turn ramping off.

The stimulation artifact removal ability of Percept^™^ PC is improved over that of previous models by blanking the amplifier during stimulation and locking the stimulation times relative to the sampling times. With a 250 Hz sampling rate, the intersample time window is 4 ms. With a pulse width of 90 *μ*s and passive recharge of less than a ms, the stimulus artifact is much shorter than the intersample time. By shorting the amplifier input briefly during the stimulation artifact, called ‘blanking’, and by timing the stimulation to arrive immediately after and time-locked to the sampling time, the stimulus artifact is greatly reduced. This enables the continuous measurement of neural signals while stimulation is on.

### 1.8. At-Home LFP recordings with Percept^™^ PC

**Figure 6.**
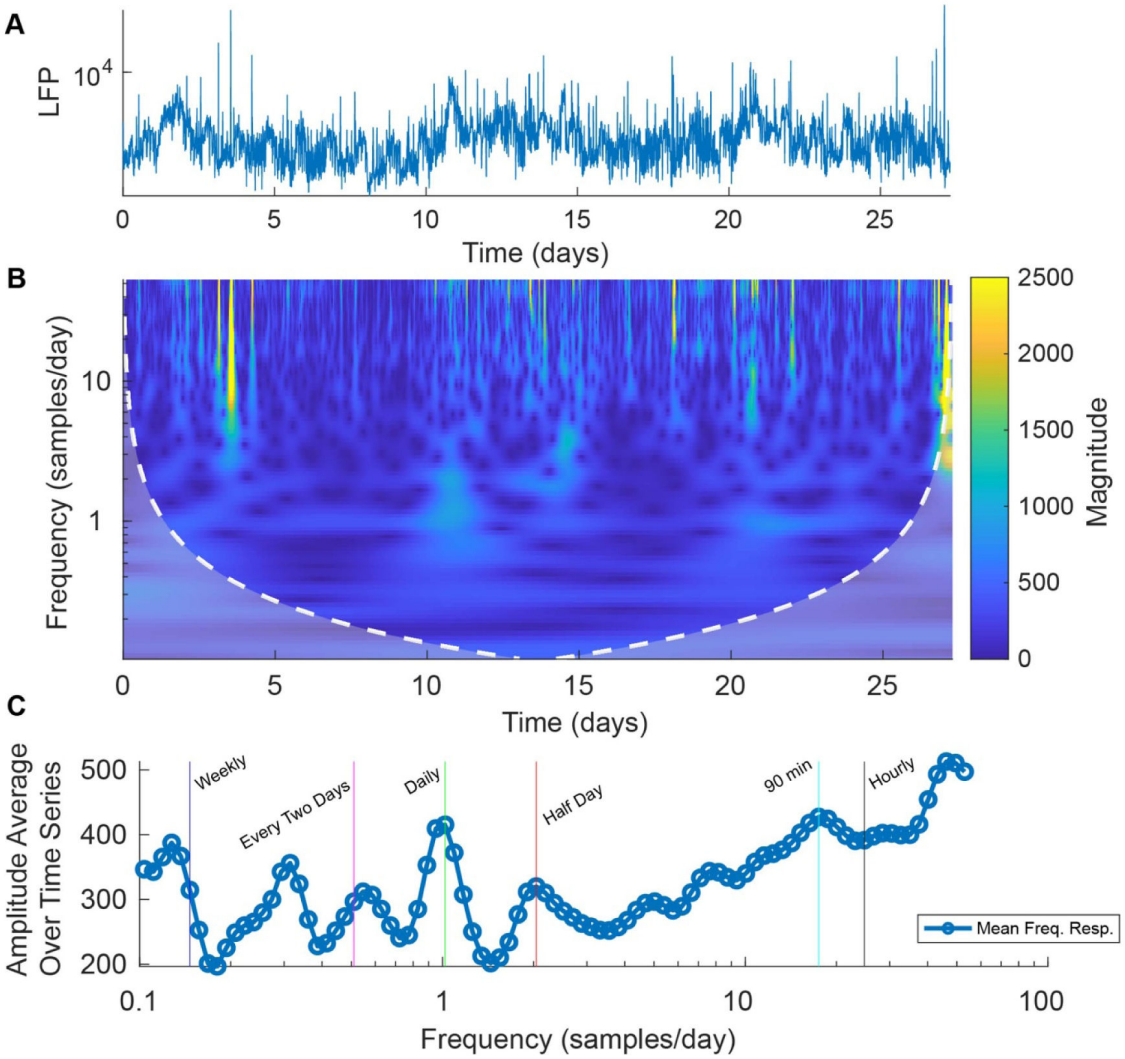
Percept^™^ PC At-home recording features (A) At-home 28 day long 10 minute-interval averaged 5 Hz wide band raw LFP power recording (Patient 001). (B) Morlet wavelet analysis of the raw at-home recording. (C) Samples/day frequency response averaged across time with the peaks of notable biorhythms marked.

In addition to the in-clinic recording capabilities, Percept^™^ PC can record a 10 min average LFP measurement within a 5 Hz band, determined by the clinician, and store it in a 60 d running memory with a first-in first out buffer. This signal allows for the detection of biorhythmic cycles [27-31] with periods of hours, days, weeks, and months; these cycles have been observed in both males and females, and in some patients, they are correlated with seizure frequency [29, 32]. The raw LFP average measurements of alpha band activity (8.79 Hz ± 2.5 Hz) over 28 d are shown in figure 6(A). The wavelet magnitude scalogram of these data shows the amplitude changes over time and frequency in figure 6(B).

Given a sampling rate of 144 samples/day, the biorhythms were defined as follows: hourly (24 samp/day), circadian/90 min (16 samp/day), half day (12 samp/day), daily (1 samp/day), every two days (0.5 samp/day), and weekly (1/7 samp/day). In figure 6(C), these cycles show clear peaks within the spectral response of these long-term recordings and demonstrate the capability of Medtronic Percept^™^ PC to capture biorhythms within at-home recordings of a particular frequency band.

### 1.9. Percept^™^ PC longevity and power consumption with stimulation parameters

Device power output and consumption capabilities should be considered when developing closed-loop neuromodulation methodologies. Many devices on the market utilize primary cell batteries with a fixed energy storage. Rechargeable battery devices are currently available from two of the three available device manufacturers and remain a popular choice with patients due to their longevity. A rechargeable battery lifespan is determined by the total allowed charge cycles while maintaining required output specifications. Here we will provide a brief description of how different waveforms affect the energy consumption from the battery.

Broadly, the device energy consumption is dependent on the energy used to deliver the therapeutic stimulation, termed Total Electrical Energy Delivered (TEED), and the hardware and software energy required for operation, such as generating the waveform and telemetry. For a single stimulation waveform, TEED can be calculated from the constant current stimulation amplitude, frequency, pulse width, cycling rate, and electrode impedance.

**Figure 7.**
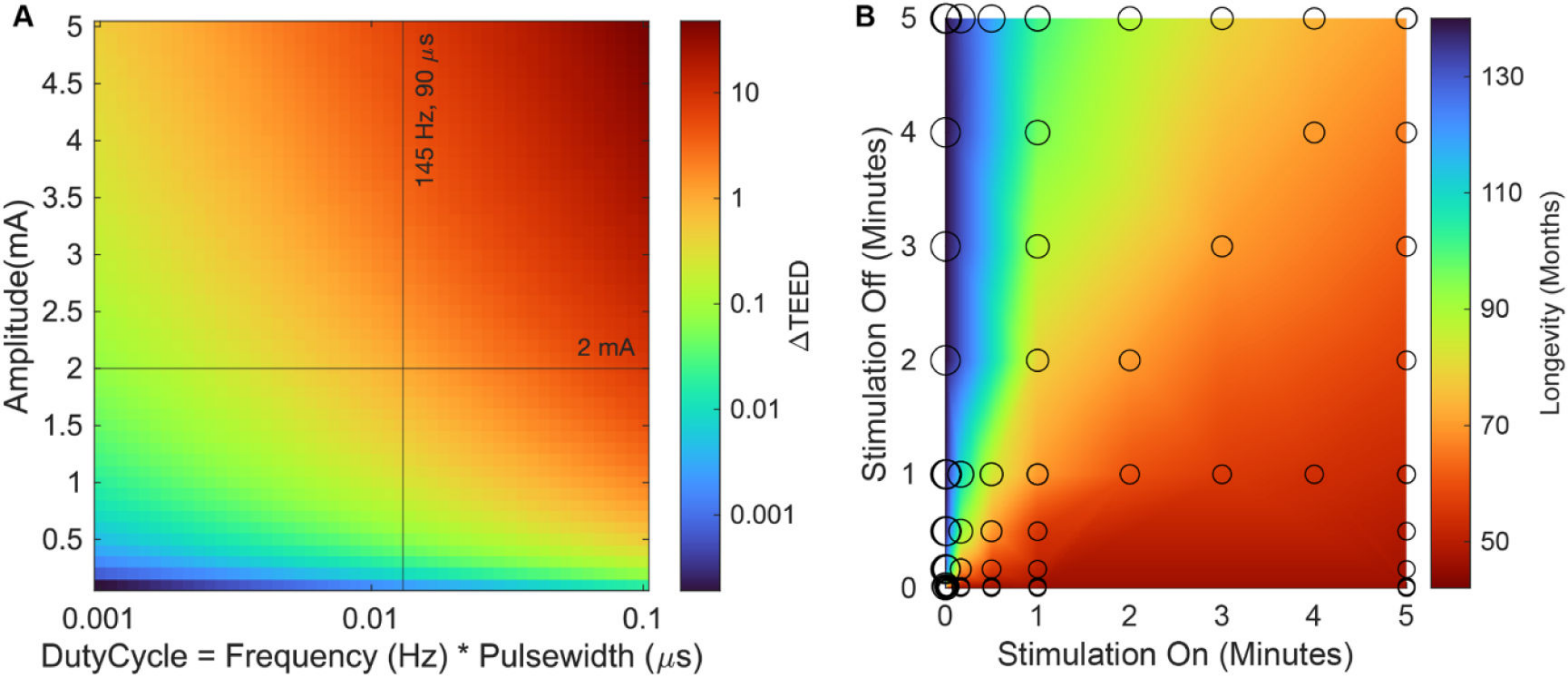
Percept^™^ PC longevity and energy delivery (A) TEED calculated theoretically from amplitude, frequency, and pulse width. TEED is shown in log/linear space relative to the TEED for SANTE trial [7] standard stimulation parameters of 2 mA, 145 Hz, and 90 *μ*s while assuming a 1000 ohm resistance. (B) Physician tablet demo mode longevity (months) calculator results using the SANTE trial [7] standard stimulation parameters of 2 mA, 145 Hz, 90 *μ*s, and a 1000 ohm lead contact impedance with cathode monopolar stimulation at contacts 1 and 2 while parameter sweeping cycling on and cycling off durations from 0.1 s to 5 min.

Energy delivered for each pulse of constant current can be calculated as [33]:

$${\text{TEED}}_{\text{wave}}={\text{Current}}^{2}*\text{Impedance}*\text{Pulse width}$$

where TEED is measured in watts, Current in amps, Impedance in ohms, and Pulse Width in seconds. In figure 7(A), TEED as a function of Duty Cycle (${\text{Frequency}}^{\;*\;}\text{Pulsewidth}$) and amplitude is theoretically calculated and plotted as a heatmap. The color illustrates relative TEED when changing stimulation parameters compared to the TEED at the standard SANTE trial parameters.

The total energy delivered over a day can be calculated as:

$${\text{TEED}}_{\text{day}}={\text{TEED}}_{\text{wave}}*\text{Frequency}*1440*\text{Cycle On}∕\;\;\times (\text{Cycle On}+\text{Cycle Off})$$

where Frequency is in Hz (pulses/second) and the proportion of the time cycling of stimulation on Cycle On∕(Cycle On+Cycle Off). Figure 7(B) shows how the device longevity is affected by the proportion of Cycle On vs Cycle Off. Total battery longevity can be estimated using tables from Medtronic’s reference document *System Eligibility & Battery Longevity* [34]. This table provides constant voltage and current stimulation energy usage (EU) tables to calculate the EU of Activa PC and SC systems given stimulation parameters, electrode impedances, usage across multiple programmed groups, and usage of stimulation within the day. While this table does not directly address Percept^™^ PC’s battery consumption, this calculation tool is helpful to illustrate how the different stimulation parameters affect the EU. Medtronic states cycling too quickly with Percept^™^ PC can be detrimental to the battery, especially when cycling faster than 1 s on and 1 s off [34]. In figure 7(B), longevity was estimated using the calculator available in the physician tablet demo mode to determine the effects of the cycling parameters on the longevity. For the heatmap, the data were then bilinearly interpolated between sampled points.

Data recording with Percept^™^ PC also impacts the device longevity. In the clinic, Percept^™^ PC can stream data for up to 4 h continuously. The sampling and streaming of neural data in the clinic are estimated to reduce the IPG battery life by 1 d for every hour of streaming. BrainSense recording at home of the 10 min averaged power within a 5 Hz band is estimated to reduce the IPG battery life by about 3% [34].

## 2. Discussion

Medtronic’s Percept^™^ PC device is an excellent tool for recording neural activity while stimulating in a wide range of DBS applications. The capability to simultaneously stimulate and record within the DBS lead-implanted region allows for in-clinic and ongoing evaluation of stimulation therapies for overall refinement and/or personalized treatments. Percept^™^ PC has been FDA approved for specific clinical use cases and was made available following Medtronic’s predecessor research device Summit RC + S. Percept^™^ PC/Summit^™^ RC + S provides improvements over the Activa PC + S such as reduced volume and weight, increased data rates, sampling rate, ADC resolution, artifact rejection, and stimulation channels [5, 12]. These features provide significant improvement over previous models while providing a good signal to noise ratio for use in many closed-loop neuromodulation approaches.

### 2.1. Percept^™^ PC capability and SenSight^™^ leads

The filtering characteristics, artifact removal capability, and lack of noise within BrainSense recordings allow for the analysis of therapies across DBS neuromodulation. Historically, neural recording during stimulation has required separate, large, and externalized recording systems that are sensitive to noise and often record at different locations than the stimulation electrodes. Medtronic’s previous Activa PS + S system contained sensing capabilities but these could not be utilized simultaneously while stimulating. Additionally, SenSight^™^ electrodes improve the signal fidelity over Legacy electrodes, and their segmented leads allow for current steering.

### 2.2. Device limitations

The BrainSense feature within Percept^™^ PC uses more power, which shortens the lifespan of the primary cell. While a rechargeable cell battery within Percept^™^ PC is expected in the future and was previously available in the Summit device, Percept^™^ PC power consumption should be a factor considered when long term monitoring and defining stimulation settings. Regardless of stimulation settings, 1 year of BrainSense monitoring use will reduce the expected life of the primary cell of Percept^™^ PC by 2 months from the expected average of 6 years [34]. The total IPG battery longevity therefore depends more on the core hardware operations and stimulation settings of amplitude, frequency, pulse width, and duty cycle, than recording at home. Adjusting stimulation parameters may result in larger power consumption for little therapeutic benefit and long durations of patient recording may be necessary for biomarker feedback given the DBS indication. While there is no clear answer to this power consumption versus recording tradeoff, these aspects will be important when designing closed-loop therapies using Percept^™^ PC.

With the limited sampling rate of 250 Hz, Percept^™^ PC is limited in detecting with adequate resolution the fast signals, such as the evoked responses, that often occur within milliseconds following stimulation. Percept^™^ PC is also not designed for long-term high-rate sampling due to the power cost and storage demands of streaming and storing large amounts of data. However, the 2 month buffer of continuously recorded 10 min average power of neural activity could be a useful biomarker for testing the efficacy of therapy.

Because of the slow sampling rates, stimulation above 125 Hz are aliased into lower frequency bands. Hardware and digital filter features minimize the impact of the aliasing into frequencies below 80 Hz for stimulation above 165 Hz.

Artifacts are seen when stimulation is on, even when the amplitude is zero, due to electronic noise in the stimulator. Thus, it is generally better to turn off stimulation than to set the stimulation amplitude to zero to minimize this artifact. Artifacts from the electronics are also seen when the amplitude is ramping up and off, which can interfere with neural signals; thus, we generally recommend turning off the soft start feature while recording neural signals in the clinic.

### 2.3. LFP monitoring in clinic and at home

Medtronic’s Percept^™^ PC allows for the at-home monitoring of frequencies in a particular window over a large frequency band. It is also equipped to adjust settings, such as the stimulation amplitude, based on the power measured for closed-loop therapies. One application currently being investigated for PD and essential tremor is to change the stimulation amplitude in concert with changes in beta power detected within the subthalamic nucleus [35, 36]. This Percept^™^ PC closed-loop therapy feature could be useful where a frequency band power measurement is a significant biomarker for therapy efficacy.

Additionally, patients have the ability to switch between up to four pre-defined stimulation therapies via their clinical monitoring device if testing alternative therapies is of interest. Throughout at-home therapy delivery, patients can record any events or side effects through one of up to 4 different event types pre-defined by the physician. This patient prompted trigger uses a 30 s snapshot of broad band LFP activity after the trigger to calculate and store a power spectral density response. Utilizing these marked events or systematically capturing these events at home may provide insight on long term therapy effects and efficacy.

## 3. Conclusions

Medtronic’s Percept^™^ PC is one of the few IPGs that can record neural signals and apply stimulation on the same lead. The noise characteristics and rejection of stimulation artifacts make the collection of neural recordings with excellent fidelity possible. The energy cost of streaming data and continuously recording data at home is minimal. Hardware and software filters are generally good at removing much of the aliased neural signals, allowing for measurements from 1–80 Hz without much artifact for any stimulation setting above 80 Hz. This device has many powerful features that could benefit biomarker discovery, closed-loop therapies, and the optimization of DBS for personalized medicine.

## Figures and Tables

**Table 1.** Clinical trial patient population (#NCT05493722).

Patient	Implanted electrodes	Lead type	Seizure location	Sex	Age
001	Bilateral	Legacy	Left Frontal Lobe	F	40s
002	Bilateral	Legacy	Bilateral	M	30s
003	Bilateral	Legacy	Multi-Focal	M	20s
004	Bilateral	SenSight^™^	Left/Central Temporal Lobe	M	30s
005	Bilateral	SenSight^™^	Multi-Focal	M	20s
006	Bilateral	SenSight^™^	Multi-Focal	M	50s
007	Bilateral	SenSight^™^	Left Parietal Lobe	M	40s

## Data Availability

The data that support the findings of this study are available upon reasonable request from the authors.

## Appendix

### Patient data used in this study

Patients in this study with focal, medically refractory epilepsy were discussed at an interdisciplinary surgical consensus conference and deemed to be good candidates for DBS. Separately, they participated in informed consented to participate in a clinical trial that was approved by the University of Minnesota Institutional Review Board (IRB) [11] (#NCT05493722). The inclusion and exclusion criteria for these patients are similar to those of the SANTE trial [7, 37]. This trial is investigating frequency band power changes in LFP recordings during and after anterior nucleus of the thalamus (ANT) stimulation for the treatment of epilepsy. Details of the patient population are shown in table 1.
